# A focus group study of healthy eating knowledge, practices, and barriers among adult and adolescent immigrants and refugees in the United States

**DOI:** 10.1186/1479-5868-11-63

**Published:** 2014-05-16

**Authors:** Kristina Tiedje, Mark L Wieland, Sonja J Meiers, Ahmed A Mohamed, Christine M Formea, Jennifer L Ridgeway, Gladys B Asiedu, Ginny Boyum, Jennifer A Weis, Julie A Nigon, Christi A Patten, Irene G Sia

**Affiliations:** 1 Department of Anthropology, Sociology and Political Science, Université Lumière Lyon 2, Campus Porte des Alpes, Bâtiment K 5, Lyon, France; 2 Department of Medicine, Mayo Clinic, 200 First St. SW, Rochester, MN 55905, USA; 3 Department of Nursing, Winona State University, 859 30th Ave SE, Rochester, MN 55904, USA; 4 Michigan State University College of Human Medicine, 1355 Bogue St, East Lansing, MI 48824-1317, USA; 5 Department of Pharmacy, Mayo Clinic, 200 First St. SW, Rochester, MN 55905, USA; 6 Department of Health Sciences Research, Mayo Clinic, 200 First St. SW, Rochester, MN 55905, USA; 7 Rochester Community and Technical College, Rochester, MN 55904, USA; 8 Center for Translational Science Activities, Mayo Clinic, 200 First St. SW, Rochester, MN 55905, USA; 9 Hawthorne Education Center, 700 4th Ave SE, Rochester, MN 55904, USA; 10 Department of Psychiatry and Psychology, Mayo Clinic, 200 First St. SW, Rochester, MN 55905, USA

## Abstract

**Background:**

Immigrants and refugees to the United States exhibit lower dietary quality than the general population, but reasons for this disparity are poorly understood. In this study, we describe the meanings of food, health and wellbeing through the reported dietary preferences, beliefs, and practices of adults and adolescents from four immigrant and refugee communities in the Midwestern United States.

**Methods:**

Using a community based participatory research approach, we conducted a qualitative research study with 16 audio-recorded focus groups with adults and adolescents who self-identified as Mexican, Somali, Cambodian, and Sudanese. Focus group topics were eating patterns, perceptions of healthy eating in the country of origin and in the U.S., how food decisions are made and who in the family is involved in food preparation and decisions, barriers and facilitators to healthy eating, and gender and generational differences in eating practices. A team of investigators and community research partners analyzed all transcripts in full before reducing data to codes through consensus. Broader themes were created to encompass multiple codes.

**Results:**

Results show that participants have similar perspectives about the barriers (personal, environmental, structural) and benefits of healthy eating (e.g., ‘junk food is bad’). We identified four themes consistent across all four communities: Ways of Knowing about Healthy Eating (‘Meanings;’ ‘Motivations;’ ‘Knowledge Sources’), Eating Practices (‘Family Practices;’ ‘Americanized Eating Practices’ ‘Eating What’s Easy’), Barriers (‘Taste and Cravings;’ ‘Easy Access to Junk Food;’ ‘Role of Family;’ Cultural Foods and Traditions;’ ‘Time;’ ‘Finances’), and Preferences for Intervention (‘Family Counseling;’ Community Education;’ and ‘Healthier Traditional Meals.’). Some generational (adult vs. adolescents) and gender differences were observed.

**Conclusions:**

Our study demonstrates how personal, structural, and societal/cultural factors influence meanings of food and dietary practices across immigrant and refugee populations. We conclude that cultural factors are not fixed variables that occur independently from the contexts in which they are embedded.

## Background

The epidemic of overweight and obesity in the United States (U.S.) is severely affecting racial and ethnic minority populations [1,2]. Latinos and non-Mexican Blacks face higher risks of obesity than non-Mexican Whites [3-5]. While obesity causes are manifold, physical activity and unhealthy eating patterns are recognized as primary factors in obesity-related morbidity and mortality [2].

The immigrant population is the fastest growing sector of the U.S. population. Recent census data shows that immigrants constitute 13% of the total population, with 53% from Latin America and 28% from Asia [6]. While recent immigrant and refugee populations may still benefit from the “healthy migrant phenomenon” [7-9] showing a better health status than native-born residents [10-12], immigrants with 15 years of residence or longer and their children are also affected by the obesity epidemic [13-15]. Furthermore, second and third generation immigrants are associated with increased body size and cardiovascular disease risk [15-19].

Much research on the obesity epidemic and associated health decline of immigrant and refugee populations in affluent countries has focused on the role of acculturation [17,20-22]. Acculturation—understood here as the process of adopting the cultural norms, and practices of the host society— has been found to be associated with increasing risk of obesity and cardiovascular disease, mediated in part by sub-optimal dietary behaviors [23]. Yet, research also underlines the limits of acculturation theory to analyze health disparities in immigrant populations [24-26]. Recent studies underscore that environmental influences (e.g., food environment [27]) and sociocultural factors (identity threat, family dynamics, cultural beliefs, socioeconomic position) underpin food preferences and dietary decisions [28-33]. Daily food rituals in immigrant families, such as meal preparation, serve as a means for parents to serve as ‘culture-bearers’ [32] while also incorporating customs of the adoptive culture more desired by the children [34]. Lifestyle and environmental changes that accompany immigration can limit options for access to health-promoting activities such as healthy diet [35].

Work on the effects of acculturation on healthy eating among immigrant populations in the U.S. has increased in recent years, with the majority of data available for Latinos. However, there is a relative paucity of data in the field’s understanding of the meanings of food and the barriers to healthy eating in other immigrant populations. People make sense of healthy eating within the multilayered context comprised of culture and tradition, mainstream population eating patterns, and ethical influences [36]. In addition, while there is ample evidence that parental control, counseling, and involvement in dietary patterns is crucial to influence adolescent eating behavior, to our knowledge, there are no studies that focus on immigrant families with data both from adults and adolescents [37]. In an effort to address these challenges, we conducted a qualitative study on the meanings of food and health in four different immigrant and refugee communities in southern Minnesota—Somali, Mexican, Sudanese, and Cambodian—which have maintained distinct communities for decades. These groups have different immigration trajectories, but all face the struggle of dietary challenges in their new country of residence due to cultural-specific obesity risk and acculturation [38-40]. For example, ethnic group affiliation may influence food choices in that ideals, identities, and roles interact with each other and the food and eating context to influence food choice [41]. Local social factors and politico-economic contexts also shape commonly shared food and migration experiences [42]. As a result, Latinos have been identified as a high-risk group for obesity [17]; South Asian dietary patterns have been linked to a higher obesity risk [43], and Somali immigrants have been observed to adopt dietary patterns in their new country of residence that puts them at risk for obesity [39]. Through focus groups (FGs) with adults and adolescents from each of these immigrant groups, we explored food preferences and beliefs and practices of dietary patterns, food environments, and barriers to healthy eating.

Typically, daily dietary practices and eating practices are implicit and subconscious, rather than deliberate and thoughtful. This study takes a social constructivist perspective [44] viewing people as actively developing meanings and understandings of the world, and drawing on their unique personal experiences and the social, cultural, and historical contexts in which they live. We used qualitative research to allow participants to reflect upon what they do and think about food and healthy eating in their own words. Qualitative research has been shown instrumental in advancing our understanding of social and behavioral aspects of food and eating [45]. A focus on the beliefs and practices and the meanings people associate with food, healthy diet, and health can enlighten culturally appropriate health intervention programs and create healthy eating environments to inform policy approaches [46].

One difficulty in designing interventions to promote healthy eating among immigrant and refugee populations is that the reasons for sub-optimal behaviors and worse outcomes are multiple, complex, and poorly understood [47,48]. Community-based participatory research (CBPR) is a way to collaboratively investigate health issues within a community, whereby community members and academics partner in an equitable relationship through all phases of the research process [49]. This is an approach to research that is particularly well suited to intervention development that addresses the interplay between health behaviors and the social determinants of health such that it helps to build community capacity, promotes understanding of culturally pertinent issues, and targets the multi-faceted barriers to health [50,51]. Furthermore, existing literature suggests that CBPR is an effective means of approaching health issues among immigrants and refugees [52,53].

Building on our existing successful CBPR partnership, this study employed FG methodology to assess 1) Ways of Knowing about Healthy Eating, 2) Eating Practices, 3) Barriers, and 4) Preferences for Intervention.

## Methods

### Community-based participatory research partnership

Our CBPR partnership began in 2004 between Mayo Clinic and the Hawthorne Education Center, an adult education center that serves thousands of immigrants and refugees per year in Rochester, Minnesota. Between 2005 and 2007, this partnership matured by formalizing operating norms, adopting CBPR principles, and adding many more community and academic partners to form the Rochester Healthy Community Partnership (RHCP) with a mission to promote health and wellbeing among the Rochester community through CBPR, education, and civic engagement to achieve health for all (http://www.rochesterhealthy.org). Since 2007, RHCP has become productive and experienced at deploying data driven programming with immigrant and refugee populations [54,55]. Community and academic partners conduct every phase of research together. In this project, community partners took leadership roles in identifying the research questions, drafting the focus group questions, recruiting participants, conducting the focus groups (e.g., bi-lingual community moderators), and in data analysis.

### Study design

The study was approved by the Mayo Clinic Institutional Review Board for the Protection of Human Subjects. We chose FGs to gain insights into shared meanings about food and wellbeing (e.g. community norms) and personal and other influences (e.g. culture, socio-economic status) related to ways of knowing about food, eating practices, and barriers to healthy eating. We chose FGs over individual interviews to reach out to a larger number of community members and allow for discussion among age and gender-specific groups. The research questions were: 1) How do immigrant adults and adolescents from Somali, Mexican, Cambodian and Sudanese communities conceptualize healthy eating? 2) What are the primary barriers to healthy eating identified by adults and adolescents in these four immigrant populations? 3) What are the immigrants’ recommendations for the development of an intervention program for healthy eating and wellbeing to overcome existing barriers? The FGs explored barriers and facilitators to both physical activity and healthy dietary patterns with the four different immigrant and refugee populations (Somali, Mexican, Cambodian, Sudanese). This paper only reports on the results pertaining to the meanings of food and barriers to healthy eating. Results pertaining to physical activity are reported elsewhere [56].

We used purposive sampling [57-59]; RHCP community partners invited immigrant and refugee families from each participating community (Somali, Mexican, Cambodian, Sudanese) to participate in FGs on healthy eating and physical activity. Over a period of six months (July-December 2011), we conducted a total of 16 FGs, including four FGs per participating community, which were homogeneous by sex and age group (four FGs each with groups of adult males, adult females, adolescent females, and adolescent males). The FGs were conducted as exploratory work to be incorporated into a planned intervention that is being designed with Somali, Sudanese, Mexican, and Cambodian immigrant families to live healthier, in federally-funded, participatory intervention study (Healthy Immigrant Families: Working Together to Move More and to Eat Well).

### Participants

The goals of the FGs were disseminated by word of mouth and presentations at RHCP meetings and at schools and places of worship. Study eligibility requirements included self-identifying as a member of one of the participating communities, being aged 11–65 years (11–18 for adolescents, and 19 and older for adults), being willing to participate in a FG with members of their community, and providing informed consent. We included both adults and adolescents, as we were interested in designing an intervention study about healthy living using the family as the unit of analysis. A majority of the adolescent participants were children of an adult who also participated in the FGs. Adults and adolescents were interviewed separately to encourage open conversation that was developmentally appropriate.

### Procedure

FGs were led by experienced moderators from participating communities who underwent FG training through the RHCP [60]. Note takers were present at each session. Linguistic concordance between moderator and participants was achieved in 13 of the 16 FGs. For the three FGs where there was discordance (Cambodian adult men and women; Mexican adult women), trained medical interpreters were utilized. FG sessions lasted 90–120 minutes each and were conducted at various community locations, including a school, a mosque, a church, a temple, and a community center. FG sessions were digitally recorded with permission, translated to English by native speakers, and transcribed. Translation integrity was verified by a native-language speaker and FG moderators reviewed transcripts for accuracy. Participation was voluntary and all participants provided oral informed consent. Food was provided at each FG. Participants received cash cards for their time.

We used social cognitive learning theory (SCT) to develop the FG guides. SCT underlines the interplay of social environmental factors (e.g. social support networks) and individual factors (e.g., self-efficacy to eat healthier) on health behavior [61]. Low self-efficacy is strongly associated with a lack of success at eating a healthy diet [62]. FG guides included questions assessing 1) participants’ understanding of a healthy diet, including soliciting descriptions of food, food environment, and dietary patterns; 2) participants’ perceived barriers to eating healthy food individually, as a family and as a community; 3) differences in dietary patterns “back home” and in the U.S.; and, 4) recommendations of how they could eat healthier. FG guides were pilot-tested with an adult from each immigrant community and refined before use.

### Qualitative analysis

FG transcripts and notes provided the data for analysis. A qualitative analysis team composed of eight RHCP academic and community partners (AM, GA, GB, CF, SM, JR, KT, MW) used a combination of qualitative content analysis [63,64] and grounded theory [65] for coding and analysis. Eight analysts performed an interpretive reading of all transcripts to identify both manifest and latent content and then developed a coding frame through discussion and consensus, and based on the frequencies of relevant categories. Once evaluated through trial coding, analysts worked in teams of two to perform the main coding on all transcripts. For final coding, each transcript was coded independently by two analysts who then met to agree on the final coding. This method was used to ensure coding reliability across analysts. Analysts then transformed the codes categorical themes and sub-themes to understand the meanings of food, health, and wellbeing among participants. Final themes and sub-themes were derived through a deliberative process among analysts. Analytic memos and data tables were derived to inform presentation of results. Analysis was facilitated by QSR International’s NVIVO-9 software (QSR International, Pty. Ltd.).

## Results

### Participants

In total, 127 people participated in 16 FGs, four from each immigrant community (Somali, Mexican, Cambodian, Sudanese), respectively. The total number of participants who participated across all 16 groups were as follows: a) Somali adolescents ages 14.3 ± 1.1 (n = 21, 43% female), b) Somali adults ages 40.2 ± 11.3 (n = 15, 47% female), c) Mexican adolescents ages 14.8 ± 1.7 (n = 22, 59% female), d) Mexican adults ages 42.8 ± 5.4 (n = 14, 50% female), e) Cambodian adolescents ages 14.6 ± 1.5 (n = 15, 60% female), f) Cambodian adults ages 58.8 ± 13.2 (n = 14, 71% female), f) Sudanese adolescents ages 14.3 ± 1.9 (n = 15, 53% female), g) Sudanese adults ages 47.7 ± 12.2 (n = 11, 45% female). Additional details about the sample are shown in Table 1.

**Table 1:** Demographics of study participants

**Focus Groups**	**N**	**Gender (% female)**	**Age (mean + SD)**	**Years lived in U.S. (mean + SD)**	**Language most commonly spoken at home (% English)**	**Mean annual household income (US dollars)**	**Education level (% high school equivalent or less)**
**Cambodian**							
*Adult (2)*	14	71%	58.8 ± 13.2	18.6 ± 10.1	0%	14,862	100%
*Adolescent (2)*	15	60%	14.6 ± 1.5	9.1 ± 5.8	0%		
**Mexican**							
*Adult (2)*	14	50%	42.8 ± 5.4	12.7 ± 7.5	0%	24,000	86%
*Adolescent (2)*	22	59%	14.8 ± 1.7	12.2 ± 3.7	27%		
**Somali**							
*Adult (2)*	15	47%	40.2 ± 11.3	11.1 ± 4.2	0%	28,092	60%
*Adolescent (2)*	21	43%	14.3 ± 1.1	11.2 ± 3.7	24%		
**Sudanese**							
*Adult (2)*	11	45%	47.7 ± 12.2	8.5 ± 4.5	9%	24,857	45%
*Adolescent (2)*	15	53%	14.3 ± 1.9	12.8 ± 2.9	33%		

### Themes

The results were highly consistent across the four communities and 16 FGs with some generational differences. Therefore, the results are summarized according to the major themes expressed across all groups with findings differentiated by age group (‘adults’ and ‘adolescents’) when differences were apparent (see Tables 2, 3, 4 and 5).

**Table 2:** Theme 1: ways of knowing about nutrition

**Subtheme**	**Adults**	**Adolescents**
Healthy Eating-Meanings	Mexican: “[Healthy eating is] not eating too many fats, eating a balanced meal. […] Balance is probably the key.”	Cambodian: “For me, I think healthy food is something that grows on trees or is from the soil.”
	Cambodian: “Eating healthy means making sure that things are cooked well. Raw food is not healthy.”	Mexican: “[Eating healthy means] not eating a lot.”
		Mexican: “Over-processed foods are like fake food or junk food.”
	Somali: “It is very good if a person eats healthy foods. His body will get the nutrients it needs.”	Sudanese: “Anything you see in the store probably isn’t fresh […] It’s not healthy, it’s not real.”
	Cambodian: “Food is medicine, it is vitamins.”	Somali: “It is better to cook your own healthy food because if you get it from […] stores, it has a lot of oil.”
		Somali: “Eating a non-healthy diet makes you lazy. […] Tired all the time, sleep through the day, not active at all.”
		Somali: “You feel lazier if you eat fattening foods.”
		Sudanese: “Let’s say you eat a donut, you will get lazier after that [than when] eating healthy stuff.”
Healthy Eating-Motivations	Somali: “When we got here, we noticed that we could get many diseases from eating that type of [junk] food.”	Cambodian: “ [Healthy eating is] good for your heart. […] You live longer. […] You don’t look fat”.
	Somali: “You lose weight and you lose fat. […] [Healthy eating], it’s good for the brain. You are able to do everything and remember everything but when you eat something unhealthy, you […] are always tired.”	Cambodian: “You live longer, healthier. […] You stay young. […] You have more energy.”
		Mexican: “Keeps you nice and strong.”
		Somali: Eating a healthy diet prevents overweight and obesity. […] Eating healthy can help you focus during school, work, it can help you stay active.”
	Somali: ““When you eat something unhealthy, you become […] always tired. […] The fat is in your body and fills your heart and clogs your blood vessels.”	
Healthy Eating-Knowledge sources	Mexican: “I get a lot of information from television. […] They are saying that breakfast is the most important meal of the day, so [for her kids] she makes them a really hearty breakfast.”	Mexican: “I talked to one of my teachers and she told me she had a heart attack because of pop.”
		Cambodian: “I learned about food from science class and from my parents, too.”
	Sudanese: “Every woman is expected to know how to cook.”	Sudanese: “I watched the movie Supersize Me and I stopped eating McDonalds.”

**Table 3:** Theme 2: eating practices

**Subtheme**	**Adults**	**Adolescents**
Family Eating Habits	Cambodian: “We just eat when we are hungry and we don’t eat when we are not hungry. That practice keeps us from worrying about dieting.”	Cambodian: “We eat a lot of fish. […] Mostly rice. […] Vegetables and rice.”
		Mexican: “Beans and rice. […] lots of oil.”
	Mexican: “With family and kids in particular, you need to set an example. The kids won’t eat vegetables if you don’t eat them […] otherwise they are content with their pizza or chicken nuggets.”	Somali: “My mom does not like us eating out. She is trying to cut us from the fast food.”
		Somali: “We might not have the right food to eat at home; we just like eat the same things over and over.”
		Sudanese: “My mom told me that people in [our country] are healthier because there is no such a thing ‘pop it in and I’ll microwave it.’ They cook every single day.”
	Sudanese: “We eat healthy […] We don’t have so much fast food where we originate from. Fast food is considered junk food. Here […] we try to make sure […] we feed [our children] homemade meals.”	
		Sudanese: “When my mom’s cooking it, all it is, is vegetables and then sometimes there’s a little bit of meat, but it’s not like all the time it’s meat.”
	Sudanese: “In Africa, we cook what the father needs, not the kids.”	Sudanese: “My dad, he plants things outside like in the summer, and then he cooks it and he eats it.”
	Somali: “My kids go to school and they learn	Sudanese: “My Dad always says, [when he] orders pizza or chicken [on weekends]: ‘you are not supposed to eat this stuff all the time because you are going to get fat, […] you are going to get sick from all the fat in the food. [And] the food your mom cooks is healthier than any food in America. You get stronger after eating it. That’s why most Africans are strong.’”
	about health and eating fruits and vegetables […], but when they come home they fill their stomach with pasta and rice.”	
	Sudanese: “Most of our Sudanese dishes are homemade.”	
	Mexican: “In my country, with my family, […] we prefer homemade food. It is better. It’s healthier, less fat.”	
		Mexican: “[We] are used to everything just being homemade.”
Americanized Eating Habits	“There is a lot of junk food places and you are tempted […] and not make your own food.”	Somali: “It seems like on every corner [there is fast food].”
		Somali: “Most of the [parents], when they came from like Africa […] they just want to be in America. […] They just want to be Americanized. They just want to be part of it. They don’t want to be left out.”
	“Back home, it was easier to get healthy food. […] Here it is easier to get unhealthy food than healthy food.”	
		Mexican: “[My] parents or whoever cooks, cooks healthy [food] but then after the healthy stuff, [I] go to [get] junk food.”
		Sudanese: “I used to like [homemade food] when I was a kid, but all this American food just changed me.”
		Somali: “My mom buys American food but she always cooks African food. But the only time I eat it is when I am really hungry.”
		Sudanese: “Here in America everything [is with] so much oil […]. It’s just instant; just put it in the microwave.”
Easy Food	Mexican: “[We eat] whatever is easiest, just go with that because […] that’s what the kids are looking for. […] What is already made, what is easiest.”	Somali: “If you’re too lazy to cook or something you just go to the store and get like…most of the time something unhealthy.”
		Mexican: “If you hang out with your friends and you are hungry, and you have three dollars, it’s easy to just go to Taco Bell or something.”
	Mexican: “[The kids] see a cantaloupe and instead of slicing and eating it, they would rather get something easier, […] opening a bag of chips and eat that instead.”	
		Somali: “If you parents are lazy to cook food, they will give you money so you can go grab something.”
		Mexican: “[When] you are going to McDonalds you are obviously not getting a salad, you are getting a hamburger.”

**Table 4:** Theme 3: barriers to healthy eating

**Subtheme**	**Adults**	**Adolescents**
Taste and Cravings	Somali: “For us, the type of food we like is the food we know.”	Somali: “So it’s like up to you. If you want to eat healthy… but you see chips… you want to buy it so badly. Like an apple, you don’t care.”
	Somali: “The goat meat seems good to us. The rice we fill with oil seems good to us. The pasta with lots of sauce seems good to us.”	
		Sudanese: “[In America], they advertise unhealthy food to look really good.”
	Somali: “What seems good to us is a large plate of food. We would fill it with food and pile on more food. Large pieces of meat, rice, oils…”	Cambodian: “You want to eat healthy but the others foods are calling for you.”
		Cambodian boys: “[At school] they have a box of salad you can pick, but I don’t think a lot of people pick salad; they prefer some fatty food over salad.”
	Mexican: “When we got here to the U.S., [my children] wouldn’t like pizza [and] burgers. Now, I can’t get them off the pizza or the burgers!”	
		Mexican: “There is a lot of hamburger places and you are tempted to not make your own food.”
	Mexican: “I get cravings for Mexican food.”	
	Cambodian: Some [of us] prefer Asian food just because [we] are used to eating it and feel like it stays in the stomach longer. Whereas, if you eat American food, you eat for a little bit and you feel hungry again.”	Mexican: “Sometimes people might prefer junk food because they think it tastes better and usually the healthier things don’t always taste good.”
		Somali: “I run for the hot cheetos.— Oh my God!”
	Sudanese: “I miss a lot of food that I’ve been eating over there. A lot. I realize that those foods back home were more beneficial to our bodies.”	
Easy Access to Junk Food	Mexican: “When you move to this country, you get used to […] buying and keeping […] cookies, chocolate, chips, peanuts for snacking for desserts and after the meal. […] It is easy, just after the meal to just open a dessert bar or chocolate. Or [when] I am hungry, then I open a bag of chips.”	Somali: “There is [a] McDonalds on every corner.”
		Somali: “When people don’t have food at home, they just take the family to McDonalds.“
		Mexican: “It’s easier to get junk food then healthy food because you just open a bag or put it in the microwave. [With] healthy food, you have to cut the lettuce, cut the tomatoes.”
	Somali: “When you come to the U.S., the food is mostly unhealthy because of, you know, the [frozen], and [in] cans, or McDonald’s or something like that, you know, junk food.”	
		Sudanese: “There is a lot of junk food in our school.”
		Somali: “Here it is easier to get unhealthy food than healthy food.”
Role of Family	Mexican: “I try to eat healthier but my husband will protest and say: ‘I want rice and beans’.”	Somali: “You can’t really tell your parents: ‘Your food, I don’t like it.’ They’ll just say: ‘Eat!’”
	Mexican: “We all love our kids but […] part of their bad habits are also our mistakes, our errors. We have taught them wrong.”	Somali: “My mom—she buys a lot of pop.”
		Sudanese: “My mom always cooks macaroni and cheese and stuff like that… meatballs and pasta […]. Americanized food.”
	Sudanese: “The family will be the victim now because the head of the family [decides what is eaten]. In Africa, we cook what the father needs, not the kids.”	
		Sudanese: “There is a lot of junk food at home because of what my mom buys.”
	Cambodian: “For instance if there are four people in the household the difficult thing about eating is, if I wanted sour soup, and someone else just wanted vegetable soup, it is really hard to please everyone.”	
	Mexican: “So for me, I [am] from a Hispanic family. It is all very social. […] Our problem is, […] we always get together and do a lot of social eating.”	
Cultural foods and traditions	Somali: “We eat […] more grain than poultry or red meat on a daily basis. We end up eating more than we need. That’s the problem.”	Somali: “[Somali food] seems to be healthy but they fry everything.”
		Cambodian: “For me I think Cambodian food is healthy but […] it depends […] because sometimes they use pig fat or pig skin and it is really fatty food.”
	Somali: “We don’t understand the food here.”	
	Somali: The food we know was better in our country. It was good and the sun would help you burn it off. The oil and the sugar were burnt off by the sun.”	
		Mexican: “Sometimes it is hard to make Mexican food healthy unless you want to […] but if you just make it, it is not going to be healthy.”
	Somali: We think that, the bigger a person is, the healthier. […] A skinny person is a sick person.”	
		Mexican: “Well [in] our culture, [food] is not too healthy. Just fat.”
	Sudanese: In Sudan, our food [has] a lot of fat in it. If there is no fat, then it is not right. That’s my culture.”	
		Mexican: [Our] parents want to raise [us] how they were raised or slightly better and they have the idea [that] […] feeding [us] what they had as a child will […] make you in their image.”
	Mexican: “[I am] never going to be able to go from: ‘We’ve eaten tortillas our entire life*,’* to [just eating] one [tortilla] a day. That’s just impossible for my family.”	
		Somali: “Usually in the Somali culture […] healthy diet is not really part of their […] routine.”
Time	Mexican: “I work and you know [my children] are not going to eat what you tell them to, they are going to eat what is easiest.”	Somali: “[Our parents] are just too busy because they work overtime and just come home and sleep. That’s basically what they do.”
	Sudanese: “Because I am doing two jobs and at the same time I got to school… you have to wake up early in the morning and prepare breakfast.”	Somali: “Me, I always used to get McDonald’s because I worked there. I never
		eat at home. I only eat at school. I come home, sleep; I rarely have time to eat at home.”
		Mexican: “We don’t have time to make [food]. Sudanese: “My mom doesn’t cook anymore because she is always at her job.”
		Sudanese: “My parents both work, so they don’t usually have time to cook on weekdays so they usually elect the easiest thing from McDonalds or something… and bring it back home.”
		Sudanese: “For our family, because our mom works a lot, […] we have to make food on our own and it’s easier to make junk than good quality food.”
		Sudanese: “My mom is always at work so then we pick the unhealthy choice like pop a pizza in the oven and it’s quicker, just after school.”
Finances	Somali: “Fruits and vegetables are very expensive […]. Pasta is cheap and […] when it is cooked, it has a lot of calories. So if [we] had more money, [we] could buy fruits and vegetables.”	Cambodian: “Fatty [food] costs less than fruits and vegetables [and] all the healthier stuff.”
		Cambodian: “Sometimes we don’t have the money to buy healthy foods.”
	Cambodian: “The only thing that [makes it] difficult [to eat healthy] is the money. When it comes to food [availability], it is not difficult.”	Sudanese: “Fast food is way cheaper than healthy food, so you go to McDonalds and get a sandwich for a dollar rather than [buying] food and making it yourself at home.”
	Cambodian: [Healthy food] is expensive. […] If you want to be healthy and eat healthy but you’re not having enough financial aid or money to purchase them then you just get what you can…buy what you can.”	
		Sudanese: “My mom doesn’t buy as much [the] healthiest food because sometimes you can’t always afford [that].”
		Sudanese: “McDonalds has the dollar menu.— Who is going to compete with that?”
	Mexican: “Everything is really expensive in the winter. Everything healthy.”	
		Mexican: “We don’t have anything to make [healthy food] with. […] Maybe the prices [of healthy food] are too overpriced.”
	Cambodian: “In the summer, we can do gardening and grow our own vegetables and we only have to buy meat. But in the winter we have to purchase [everything].”	
		Somali: “Healthier foods tend to be more expensive at grocery stores.”
	Sudanese: “[The hard things about eating healthy in [this town] are […]: Finance, finances!”	

**Table 5:** Theme 4 preferences for intervention

**Subtheme**	**Adults**	**Adolescents**
Family Counseling	Mexican: *“*[It would be good] if [I] had more information […] to bring […] home […]. Kids are used to eating a very certain way and any time [I] change it [I] get a lot of push back.”	Mexican: “Talking to the younger kids because they’re like the future generation and just letting them know.”
		Mexican: “A lot of people don’t listen. Maybe you can get a group to go around to people’s houses and ask if they’re healthy.”
	Mexican: “With family and with kids particularly, you need to set an example. The kids won’t eat the vegetables if you won’t eat them.”	
	Somali: “That’s part of the advocacy, that, we have to advocate to the families and the kids. Then they will know what healthy is, but […] we have to write down those tools and translate it, and acknowledge the parents also.”	Sudanese: “It’s better to do it with your family than to do it alone because if it’s hard going on […] a diet and eating healthy when your brothers and sisters are watching TV and eating junk food, and if you’re a family, [you] motivate each other: ‘Come on, you can do it!’ you know, encourage each other, other than you doing it by yourself.”
	Sudanese: “For me it’s the women in the house… […] if we need a solution the woman is a big part of that solution […] because they can change a lot of things.”	
	Somali: “People they don’t have any knowledge, especially most people who immigrate here and you know, they just have, you know, lack of knowledge.”	
		Somali: “Parents can start getting better food in the house.”
	Mexican: “Not buy the foods that are bad for you, just not buy them, not even have them at home. Just buy veggies and foods that are good for you.”	
Community Education	Mexican: “[Education at] church and things like this and together (*juntos*), or in the Mexican stores […] if you want to reach [us] you’re going to have to go to the Catholic Church.”	
	Sudanese: “We just [need] knowledge… Yep, about the benefits [of] eating healthy. Like lower the salt [content], lower these oils, sweets. […] In our community you cannot tell somebody “this food is not good” they are going to be mad. […] You cannot talk about my food. That’s cultural law.”	
Healthy Traditional Meals	Mexican: “[So that I] can learn how to incorporate the same ingredients but in more, in healthier ways, so that [I am] not changing their diet entirely, just kind of recombining.”	Somali: “If [parents] see you’re getting big because of [traditional food] they give you they’ll have to change it up or like give you smaller amounts.”

All participants reported various ways of knowing about food, eating practices, and barriers to healthy eating. Participants easily identified what they perceived as major differences between their dietary practices in their countries of origin and as immigrants to the U.S. associated with an “American way of life”. Across all 16 focus groups, including age groups, participants mentioned mainstream nutrition discourses and general knowledge of the benefits of healthy eating for disease risk reduction and better health. There was significant interaction between the domains of knowledge (ways of knowing about food) and practice (eating practices) as participants applied their understandings of these concepts to their own practice and to make sense of barriers to healthy eating.

#### Theme 1. Ways of knowing about food

All participants across the four communities consistently reported key ways of knowing about food and wellbeing with some generational differences outlined below. For Theme 1, we identified three subthemes: Meanings, Motivations, and Knowledge Sources (see Table 2).

##### Meanings

Adult and adolescent participants across all four communities agreed that a healthy diet meant eating a balanced meal. Participants across age groups referred to fruit and vegetables as important parts of a healthy diet. There was consensus that fat and oils should be avoided, and that junk food is unhealthy and “makes you tired,” and “lazier”. Fast food and processed foods as well as fats and oils were described as unhealthy food. Examples are “food is medicine, it is vitamins”, the “body will get the nutrients it needs,” “eating a balanced meal. […] Balance is the key”. Participants also underlined that they were aware that portion size affected wellbeing, and thatsmaller portion sizes were better than large ones. Somali adults explicitly noted however that big portions were of cultural importance for them.

##### Motivations

Participants consistently mentioned one motive to healthy eating was to “live longer, healthier,” “have more energy,” and avoid adverse health effects and food related diseases, such as heart attack, diabetes, being overweight. For example: “My teacher […] told me she had a heart attack because [of] pop.” Several youth mentioned junk food as unhealthy food: “you can get many diseases from eating that type of [junk] food.” Similarly, a Somali adult explained: “when you eat something unhealthy, you become […] always tired. […] The fat is in your body and fills your heart and clogs your blood vessels.” Most participants recognized that “healthy food protects you from getting sick”, and “eating healthy prevents overweight and obesity”. Participants in both age groups also described additional advantages of eating a healthy diet that extended beyond the biomedical to the psychosocial (e.g., enhance self-confidence and happiness; lower stress). Adolescent and adult participants agreed that eating healthy makes you “have more energy,” “you stay younger”, improve self-esteem, and “it can help focus during school.” Related to this, it was important for some participants to eat healthy so that they could be a role model for other family members. For example, some adults reported wanting to be a role model for their children to eat healthier. Similarly, several adolescents talked about wanting to positively influence their parents’ eating practices, which they described as unhealthy.

##### Knowledge Sources

All participants mentioned how their knowledge about food was derived from a variety of sources including schoolteachers, parents, health professionals, food labels, and the media. Most adult participants referred to television as a knowledge source, and mentioned what they had learned about food in their home country; they also spoke of health professionals as sources of knowledge. For adolescents, their parents and teachers, in addition to food labels and documentaries about the food industry, were important knowledge sources about healthy eating. For example, a Cambodian adolescent said: “I learned about food from science class and from my parents, too”. A Sudanese youth said: “I watched the movie ‘Supersize Me’ and I stopped eating McDonalds”.

Another frequently mentioned way of knowing about food was related to the role of women in food preparation at home, identified by both adults and adolescents equally. Men and women participants reported that a woman is “in charge of the food in her family” and “every woman is expected to know how to cook”. As a result, adults and youth saw women as “part of the solution” when discussing how they could make their diets healthier. One Mexican woman reported that she learned on television that “breakfast is the most important meal of the day,” so she makes sure to prepare a “hearty breakfast” for her family to start the day.

#### Theme 2. Eating practices

Participants across the four communities and age groups described their eating practices in their country of origin and in the U.S. They specifically identified a change in eating practices from one generation to the next as they became more Americanized with increasing years of residence in the U.S. Three subthemes: Family Eating Practices, Americanized Eating Practices, and Eating What’s Easy are described, separated by age group in Table 3.

##### Family practices

The family was identified as the center for learning eating habits. Ways in which participants described family eating practices marked generational differences between adults and adolescents. Virtually all participants, adults and adolescents, listed the types of food they usually ate at home, highlighting fresh or homemade food as an important part of their diet and emphasizing the importance of keeping up or respecting traditional ways of eating (which was not always deemed the healthiest food). Here, adults described how they “[didn’t] have so much fast food where [they] originate from”, and highlighted how they preferred “homemade food”. In this context, some adults re-emphasized that they wanted to be a role model for their children and showed concern about the availability of junk food in the U.S. Then again, other adults highlighted that they mostly fed their children ethnic food (“Pasta and rice”) even though their children learned about fruit and vegetables at school. Here, Mexican and Somali women underlined that they served “what the father needs”, not catering to the children’s healthier preferences for salad or vegetables. Cambodian adults specifically underlined that they avoided overeating to stay healthy in this country, where they lived a sedentary lifestyle.

Adolescent groups echoed their parents’ concerns about staying and eating healthy through balanced meals at home. All adolescents emphasized the importance of homemade and fresh food at home, distinguishing it from fast food and junk food in the U.S. Examples include: “My Dad says […] the food your mom cooks is healthier than any food in America. You get stronger after eating it. That’s why most Africans are strong;” “My mom does not like us eating out. She is trying to cut us from the fast food;” “My dad, he plants things outside like in the summer, and then he cooks it and he eats it”. Still, adolescents also had concerns that their parents served them the same (unhealthy ethnic) food “over and over again”. Somali youth especially complained that they were given “large portion sizes” and “lots of oil.” Here, adolescents agreed that the ethnic food served at home was not always healthy and had hidden oil or fat. They emphasized that they were expected to eat ethnic food when served at home, insinuating that they ate out of respect for their parents and to keep with their cultural traditions, despite their knowledge that greasy food made them unhealthy. This was an issue for both Mexican and Somali youth.

##### Americanized eating practices

Across the 16 FGs, adolescent and adults all described their assimilation into fast food culture due to environmental and structural factors. For instance, many described the abundance of junk food and fast food restaurants in the U.S., highlighting that one was “tempted not to make your own food”, and that it seemed “easier to get unhealthy food than healthy food”. One participant described how “all this America just changed [him]”. In addition, adolescent participants deplored the quality of American food. One participant said: “Here in America everything [is with] so much oil […]. It’s just instant; just put it in the microwave”. Nonetheless, adolescent participants also confessed that they had developed a taste for junk food and some even described their parents as wanting to assimilate an American way of life and the dietary changes made them feel more integrated: “they just want to be Americanized”.

##### Eating what’s ‘easy’

This subtheme intersects with the Americanization of eating practices. Adult and adolescent participants described “easy food” as a new eating habit, mentioning that they ate “whatever [was] easiest”. While adults often described their children as preferring “what’s easy,” what does not have to be cut or prepared, adolescent participants also described how their parents were sometimes “lazy” and instead of preparing food at home, would give them money to eat out. There was great variation across the groups with contradictions between the discussion about ethnic food and homemade meals, and the abundance of junk food and fast food and preference for the latter, due to different types of barriers. For example, Sudanese mothers seemed committed to preparing homemade food for their families to pass on the knowledge about traditional meals and to teach their children traditional ways of cooking. Most adults and adolescents emphasized a preference for what’s easy due to time constraints with school and work.

#### Theme 3. Barriers to healthy eating

The third theme, Barriers to Healthy Eating, was discussed at length in all FGs. Results were consistent across all four communities, gender and age groups. Self-reported barriers to healthy eating ranged from personal (taste and cravings), to societal/cultural (easy access to junk food; cultural traditions) and structural (finances; time) obstacles (see Table 4).

##### Taste and cravings

A major barrier to healthy eating, identified by both adults and adolescents, was cravings for unhealthy food, such as chips and fatty food or traditional types of food with lots of oil. Both age groups recognized that healthy eating is an individual’s responsibility but personal preference and family eating habits made it hard to stop eating unhealthy food. One adolescent said: “So it’s like up to you. If you want to eat healthy…but you see chips… you want to buy it so badly. Like an apple, you don’t care”. Another mentioned: “You want to eat healthy but the other foods are calling for you”. Other barriers were differences in the conception of ‘good’ food, as in food that is ‘good for you’ (“In America, they make unhealthy food look really good”). As noted prior, ethnic food cravings were an important topic among immigrant adults. They mentioned how traditional food was considered ‘good’, albeit not necessarily healthy according to American standards (Somali adult said: “For us, the type of food we like is the food we know;” “The goat meat seems good to us. The rice we fill with oil seems good to us. The pasta with lots of sauce seems good to us.”). Other barriers mentioned were changing tastes since coming to the U.S. and, as foreshadowed by the discussion about easy food, a higher prevalence of cravings for unhealthy food such as burgers and fries. Adults also highlighted their concern for their children, and teens highlighted their concern for a parents’ tastes for sugary soda.

##### Easy access to junk food

Along with changing preferences, the Americanization of the palate, and cravings for unhealthy food, the easy access to junk food was identified as a major barrier to healthy eating by both adults and adolescents. Examples are: “There is [a] McDonalds on every corner;” “When people don’t have food at home, they just take the family to McDonalds;” “It’s easier to get junk food than healthy food because you just open a bag or put it in the microwave. [With] healthy food, you have to cut the lettuce, cut the tomatoes”. In adult and adolescent FGs, dietary changes were observed. For instance, schools were also identified as a source of easy access to junk food, and grocery shopping of processed or canned food and snacking were described as new dietary practices. One Mexican adult said: “When you move to this country, you get used to […] buying and keeping […] cookies, chocolate, chips, peanuts for snacking for desserts and after the meal. […] It is easy, just after the meal, to just open a dessert bar or chocolate. Or [when] I am hungry, then I open a bag of chips”.

##### Role of family

While the family was described as the nexus of their dietary practices and a potential facilitator for healthy eating or dietary change, the family was also viewed as an obstacle to healthy eating. Here, adult and adolescent groups identified family members and the transmission of unhealthy eating practices as a major barrier to healthy eating. Examples are: “I try to eat healthier but my husband will protest and say: ‘I want rice and beans’;” “We all love our kids but […] part of their bad practices are also our mistakes, our errors. We have taught them wrong;” “There is a lot of junk food at home because of what my mom buys”. Intergenerational variation in taste in the same family was identified as a barrier to eating healthy for youth who professed wanting to change to a diet based on fruit and vegetables and low in carbohydrates and fat. Furthermore, youth identified social eating as a family barrier (“So for me, I [am] from a Hispanic family. It is all very social. […] Our problem is, […] we always get together and do a lot of social eating”). As noted earlier, youth felt compelled to eat what the parents would served them even if they knew it was unhealthy. One Somali youth said: “You can’t really tell your parents: ‘Your food, I don’t like it.’ They’ll just say: ‘Eat!’”.

##### Cultural foods and traditions

This subtheme intersects with taste and cravings for unhealthy food. Many adult participants reported the transmission of a traditional high fat and high carbohydrate diet from the home country because they are familiar. Examples are: “Well [in] our culture, [food] is not too healthy. Just fat;” “We don’t understand the food here;” “[I am] never going to be able to go from: ‘We’ve eaten tortillas our entire life,’ to [just eating] one [tortilla] a day. That’s just impossible for my family.” With respect to eating practices, participants mentioned “eating more than we need” and serving big portion sizes. While all communities across gender and age underlined that traditional food contained a lot of fat and oil (“If there is no fat, then it is not right. That is my culture.”), body size ideals were also discussed in this context. In particular, Somali participants especially underlined the importance of big portion sizes and the image that “a bigger person is healthier” and “a skinny person is a sick person”.

##### Time

Lack of time for adequate food preparation of homemade and fresh food was one of the primary structural barriers identified across all communities, age and gender groups. As noted in conjunction with *Eating what’s easy*, and *Easy access to junk food,* there was consensus that preparing a healthy diet takes time and school and work schedules impeded dedicating enough time to preparing healthy food. Examples are: “Because I am doing two jobs and at the same time I go to school… you have to wake up early in the morning and prepare breakfast;” “I never eat at home. I only eat at school. I come home, sleep; I rarely have time to eat at home;” “We don’t have time to make [food];” “My mom doesn’t cook anymore because she is always at her job”.

##### Finances

All participants reported the high cost of healthy food as another important structural barrier to eating healthy meals. A Cambodian adult said: “[The hard things about eating healthy] in [this town] are […]: Finance, finances!” Among adults, limited finances were reported as a major hurdle to feeding the family a healthy but filling meal (“The only thing that [makes it] difficult [to eat healthy] is the money.”). Youth identified their parents’ good intentions but limited ability to provide healthy food for them (“My mom doesn’t buy as much [the] healthiest food because sometimes you can’t always afford [that]”). Cheap fast food came up as a primary topic to allow families to eat while staying on a budget. As one youth stated: “McDonalds has the dollar menu.— Who is going to compete with that?” The high price of fruit and vegetables were noted as a hurdle to healthy eating year-round, even if they talked about cutting their costs by harvesting from their vegetable garden over the summer.

#### Theme 4. Preferences for interventions

All FGs discussed ideas about how to design a health promotion program with the goal of improving healthy eating. Unanimously, adults and adolescents highlighted the importance of group programs to reach out to families and to the entire community. As noted in the themes before, adults and adolescents viewed their eating practices within the context of their community and their family. As a result, they emphasized the importance of educating their entire family and organizing community activities to support dietary change toward healthier meals. Quotes are separated by age groups in Table 5.

##### Family counseling

Adults and youth reported their recommendations to develop a program for family healthy eating counseling in order to change eating practices and reduce the barriers to healthy eating. Examples are: *“*[It would be good] if [I] had more information […] to bring […] home […]. Kids are used to eating a very certain way and any time [I] change it [I] get a lot of push back;” “It’s better to do it with your family than to do it alone because it’s hard going on […] a diet and eating healthy when your brothers and sisters are watching TV and eating junk food, and if you’re a family, [you] motivate each other: ‘Come on, you can do it!’ you know, encourage each other, other than you doing it by yourself.” Learning how to shop for healthier food was also mentioned as an important subtheme in the context of family counseling.

##### Community education

Adult and adolescents across community and gender groups identified the community as a main site for healthy eating education. Group-based intervention was deemed a desired approach given the habit of social eating for religious and cultural holidays and emphasizing the importance of the cultural transmission of traditional ways of eating. Togetherness was frequently mentioned (at the mosque, the church, at school). For example: “[Education at] church and things like this and together (*Juntos, a tutoring program for children and their parents*), or in the Mexican stores […] if you want to reach [us] you’re going to have to go to the Catholic Church.” Adult participants suggested that educational material should be framed in terms of knowledge about specific unhealthy food, rather than targeting traditional food as unhealthy (“We just [need] knowledge… Yep, about the benefits [of] eating healthy. Like lower the salt [content], lower the oils, sweets. […] In our community you cannot tell somebody ‘this food is not good’ they are going to be mad. […] You cannot talk about my food. That’s cultural law”). Almost echoing these concerns, adolescents viewed the community as a prime locus for intervention, along with the family, in order to address the challenges of having to continue a cultural tradition of unhealthy food or big portions they had described experiencing in their homes and in social settings.

##### Healthier traditional meals

Women adult participants reported a preference for education on how to make their traditional meals healthier by switching to healthier ingredients but without losing taste. Somali and Mexican women professed not knowing how to substitute unhealthy ingredients in their traditional meals with healthy ones or complained about their husbands not wanting them to change anything from the traditional ways, making it harder for them to adopt a dietary change toward lighter foods that better fit a sedentary lifestyle in the U.S.

As noted before in the context of family barriers to healthy eating, adolescents mentioned that their parents should serve them healthier meals, especially when they noticed that the traditional meals or large portion sizes contributed to them gaining weight. “If [parents] see you’re getting big because of [the traditional food] they give you, they’ll have to change it up or like give you smaller amounts”).

## Discussion

This FG study is one of the first to qualitatively explore the ways of knowing about food, dietary practices, and barriers to healthy eating that targeted both adults and adolescents across multiple immigrant and refugee communities in the U.S. Our study examined the generational, gender, and ethnic differences as observed through self-reported knowledge and practices of dietary patterns in four immigrant and refugee communities: Somali, Sudanese, Mexican, and Cambodian. Overall, our findings indicate that these immigrant communities arrive with healthy and unhealthy ways of knowing about eating, and they adapt both healthy and unhealthy patterns of eating from their new country.

Prior research suggested that the ‘healthy immigrant phenomenon’ declines with longer residence and increased assimilation into U.S. society, leading to poorer health outcomes such as obesity and chronic disease. Our study provides mixed support that acculturation among immigrants is associated with dietary change toward less healthy eating patterns. Our results confirm that the process of acculturation is multidimensional and complex [66]. Concordant with the findings of previous studies [45], we found that immigrant dietary patterns are influenced simultaneously by the family, transitional events, socioeconomic context, food components, food production methods, physical outcomes, psychosocial outcomes and personal goals.

We found that generational differences create a divide between how immigrant adults and adolescents conceptualize and describe their dietary practices. We specifically observed different perspectives of immigrant adults who came with set food preferences, and immigrant youth who have spent most of their forming years in the U.S. The differences in perspective between adults and adolescents indicate that dietary change toward an Americanization of immigrant and refugees communities is different between generations and that reconciling these differences may be burdensome for families. Adolescents may view their taste for Americanized food as a point of contention with their parents who insists on more traditional food ways. These findings confirm previous studies that highlighted how eating patterns are influenced by family members, indicating that food decision-making within the family unit is a multidimensional process [67]. The findings further confirm studies showing that adolescent intake of fast food is positively correlated with parents who attempt to directly control access to and intake of food [68]. Here, our findings on knowledge and food practice were contradictory, indicating that while immigrant youth tend to develop a taste for fast food and “easy food,” they also develop a greater awareness that fruits and vegetables are healthier options than what is frequently consumed. A plausible explanation of this contradiction relates to the structural barriers to healthy eating identified in this study, indicating that the association between acculturation and dietary change in immigrant groups varies within particular age groups. Further, these findings confirm that the food environment and sociocultural factors, such as identity threat, family dynamics, cultural beliefs and socioeconomic position underpin food preferences and dietary patterns and influence dietary change [28,29,31]. In this context, our study suggests that cost and high workload of parents lead to significant barriers to healthy eating and recourse to “easy food,” similar to other studies which found that eating patterns are less structured in families of children who are obese [69].

In sum, our study indicates that culture is not a sole determining variable for all immigrant groups and highlights contextual variations, especially between adult and adolescent groups. Indeed, these generational differences were far more significant than the small differences observed between ethnic groups. Finally, our study supports a role for community-based interventions to improve dietary practices among immigrant families [70].

### Limitations

There are several limitations concerning our sampling technique and methodology. We conducted four FGs in each community with only one per gender/age group. In a larger study, we would have conducted more FG per age/gender group to increase our sample size. In addition, not all FGs were conducted by native language speaker moderators. This resulted in longer sessions for one Mexican and two Cambodian FGs, contributing potentially to loss in time to go deeper into the different topics. In addition, where language concordance was not achieved, the moderators may have been less familiar with the respective communities, which may have influenced the follow up questions. Further, not all participants contribute equally in all FGs, potentially leaving shy or withdrawn participants out. To avoid this bias, a longer study time frame would have allowed us to conduct individual interviews in addition to FGs. Finally, this study analyzed self-reported food preferences and eating practices. The larger study timeframe did not allow sufficient time for gathering observational data of actual health behaviors prior to developing the intervention program. Future studies of observable immigrant family eating practices are needed to deepen our understanding of the eating preferences and self-reported eating patterns in these immigrant and refugee populations.

## Conclusion

To conclude, our study confirms the limits of acculturation theory to analyze health disparities in immigrant populations [24-26], and demonstrates how the process of acculturation is complex and multidimensional [66]. Likewise, our results suggest that the erosion of the ‘healthy immigrant phenomenon’ is not based only on acculturation. This also means that cultural factors are not fixed variables that occur independently from the contexts in which they are embedded. Further, our study highlights the importance of intergenerational variability as it relates to immigrant eating practices and dietary change. Overall, our findings indicate that personal, structural, and societal factors work together to shape the food preferences and practices of Somali, Mexican, Sudanese, and Cambodian immigrant populations in the U.S.

As implications for practice, we suggest that health promotion programs for these four immigrant populations should focus on families as units of intervention rather than individuals in order to address inter-generational differences observed. Future studies are needed with diverse immigrant and refugee populations to provide an in-depth analysis of the perspectives and self-reported eating practices observed herein.

## Competing interests

The authors declare that they have no competing interests.

## Authors’ contributions

KT participated in data analysis and drafting of the manuscript. MW participated in design of the study, data analysis and drafting of the manuscript. SM participated in design of the study, data analysis and drafting of the manuscript. AM participated in design of the study, data analysis and drafting of the manuscript. CF participated in data analysis and drafting of the manuscript. JR participated in data analysis and drafting of the manuscript. GA participated in data analysis and drafting of the manuscript. GB participated in data analysis and drafting of the manuscript. JW participated in design of the study and drafting of the manuscript. JN participated in design of the study and drafting of the manuscript. CP participated in design of the study, data analysis and drafting of the manuscript. IS participated in design of the study and drafting of the manuscript. All authors read and approved the final manuscript.
